# Role of Online Retailers’ Post-sale Services in Building Relationships and Developing Repurchases: A Comparison-Based Analysis Among Male and Female Customers

**DOI:** 10.3389/fpsyg.2020.594132

**Published:** 2020-12-23

**Authors:** Muhammad Kashif Javed, Min Wu, Talat Qadeer, Aqsa Manzoor, Abid Hussain Nadeem, Roger C. Shouse

**Affiliations:** ^1^Sichuan University, Chengdu, China; ^2^Department of Economics, Sapienza University of Rome, Rome, Italy; ^3^Khawaja Fareed University of Engineering and Technology, Rahim Yar Khan, Pakistan

**Keywords:** product return, product exchange, product maintenance, customer satisfaction, trust, repurchase intention

## Abstract

Customers are skeptical about shopping online because e-commerce environments are typically considered impersonal. To assure product quality and to enhance customer proclivity in such environments, post-sale services (i.e., product returns, exchange, and maintenance) may be considered to alleviate customers’ skepticism. Therefore, this study’s objective is to investigate the role of an online retailer’s post-sale services (i.e., product return, exchange, and maintenance) on customers’ attitudinal (building relationships) and behavioral aspects (developing customers’ repurchase intentions). Structural equation modeling is applied to data collected through an online survey answered by 409 online customers of jd.com (after missing data were removed). Research findings show that product return, exchange, and maintenance services are strongly predictive of online customer satisfaction, and satisfaction significantly impacts customer trust. Both customer satisfaction and trust, as indicators of relationship quality, further mediate the links between product return, exchange, and maintenance services and online customer repurchase intention. In addition, differences between male and female customers were found in various aspects of online retailers’ product return, exchange, and maintenance services. This is the first empirical study that not only examines the influence of all three dimensions of online retailers’ post-sale services on customers’ online shopping perceptions and decisions, but also considers differences between male and female customers. Finally, this research provides theoretical and managerial implications based on conceptual and empirical evidence.

## Introduction

The online shopping environment is considered complex and competitive (Javed and Wu, 2020). A recent study revealed that consumers are skeptical about using online channels (Lu et al., 2018). Accordingly, estimates of shopping cart abandonment rates range to more than 50% of transactions (Baymard Institute, 2019; Bell et al., 2020). More importantly, retailers face difficulty in retaining customers who frequently switch to other online retailers (Jain et al., 2017; Kumar et al., 2018). Compared to offline competitors, online businesses face more negative consequences including online retailers’ credibility, inability to inspect the product before receipt, and the physical distance between buyer and seller (Davari et al., 2016). In this environment, online retailers’ post-sale services may reduce customers’ pre-purchase uncertainty (Heiman et al., 2001; Ramanathan, 2011). For example, consumers’ psychological concerns will be relieved if they know that they can easily exchange, return (Hong and Cha, 2013) or repair of any purchased product. Yet, it is unclear how important post-purchase activities are to e-commerce and its growth (Cao et al., 2018). Thus, marketers and companies need to understand consumers and their relevant behavior in online shopping systems (Hussain et al., 2020).

Post-sale services are considered one of the most notable factors in sustaining consumers and directly influence customers’ value perceptions (Chen et al., 2017). In the online shopping context, post-sales activities are quite recent (Frasquet et al., 2016) because customers’ attitudes toward online shopping change over time as they become more familiar with the online shopping environment (Moriuchi and Takahashi, 2016; Rai et al., 2018). Accordingly, an internet retailer’s quality of service offerings can enhance online customers’ loyalty and enable retailers to determine customers’ new demands (Yu et al., 2015). Despite the importance of post-sale service, it has received less attention from researchers as compared to pre-sale services (Cao et al., 2018; Javed and Wu, 2020; Lamba et al., 2020). Researchers note that while retailers offer higher levels of access and transaction convenience (i.e., pre-sale services), customers continuously experience difficulty with post-sale procedures (Lai et al., 2014). More research is thus needed to investigate the role of online retailers after sale stages in relationship and repurchase development.

Research reveals that inefficient post-sale services may lead to online customers’ retaliation (Arruda Filho and Barcelos, 2020). Hassle-free order cancelation and easy return and refund procedures have become critical task-related expectations and important indicators of service excellence (Singh, 2019). Understanding the influences of product returns on customer loyalty is therefore essential for online retailers (Griffis et al., 2012). However, knowledge concerning if and how product return experiences actually effect customer repurchase intention or loyalty is limited (Griffis et al., 2012). Thus, online shopping policies regarding product return are increasingly coming under inspection, and Davari et al. (2016) call attention to return policies to understand their role in online retail repurchase intention.

Beyond product return, the customer’s ability to exchange a purchased product has also become an increasingly important issue for e-commerce management. The implications of product exchange differ from those of cash refund, as the latter offers less opportunity for continued customer interaction (Han et al., 2017). More importantly, researchers also noted that a retailer’s post-purchase policies such as product exchange have a positive influence on customer retention (Minnema et al., 2018). However, the impact of online retailers’ product exchange services in an empirical model, as a single construct, with respect to the customer-retailer relationship and customer purchase behavior has remained unexplored so far.

In addition, repairs or maintenance are becoming a key concern especially for those who shop online. For example, home appliances, and electronic items purchased online may require repairs during the warranty period. Moreover, Owen and Mobin (2015) noted that in recent years products have become increasingly complex, thereby increasing the buyer’s after-sale risks. Therefore, maintenance or repair of such products is becoming an increasingly significant aspect due to their progressively more sophisticated technologies (e.g., electronics). To fill this void in the literature, Javed and Wu (2020) recommended that future research should consider and examine the role of repairing or maintaining a purchased product on customer purchase decisions as an important post-sale service element for online retailers.

In addition to online retailer’s post-sale service influence on customer purchase behavior, prior empirical research on product return has focused on topics such as customers’ perceptions of the product return policy (Pei et al., 2014; Hjort and Lantz, 2016; Janakiraman et al., 2016; Jeng, 2017; Oghazi et al., 2018; Yan and Pei, 2019), product return time leniency (Rao et al., 2018), and consumer response to denied product returns (Dailey and ÜLkü, 2018). In a recent study, Javed and Wu (2020) examined the role of an online retailer’s post-sale services using three items (i.e., refund, return, and exchange) as a single construct, arguing that future studies should analyze product exchange, return, refund, and repair offers as different dimensions affecting customer e-satisfaction, e-trust, and intent to repurchase. The current study helps fill this gap.

Moreover, in the context of e-commerce, gender differences in customer behavior do exist, varying with the customer group, environment (Kim et al., 2007; Wang and Kim, 2019), and in the relative influence of each antecedent (Lin et al., 2019). For example, it is suggested that male customers are more rational, whereas females are more emotional (Wang and Kim, 2019). Literature has further revealed that unlike male customers, females prefer higher levels of convenience (Lai et al., 2014), easy accessibility and ease of use (King, 2009). Wolin and Korgaonkar (2003) revealed that male and female customers have diverse psychological pre-dispositions toward online purchases, thus supporting the idea that gender will affects buyers’ preferences toward online shopping behavior and decisions. Accordingly, gender differences have been studied in numerous e-commerce domains. However, in the context of online retailers’ post-sale services (product return, exchange, maintenance, etc.), the literature on gender differences in building a buyer-seller relationship and developing repurchases is limited. Hence, it is another objective of this study.

## Theoretical Foundation and Hypotheses Development

Prior researchers have considered product exchanges, returns, and financial refunds as distinct components of post-sale service (Grewal et al., 2004; Kalia, 2017). Ramanathan (2011) suggests, however, that an analyzed product return/refund is one variable. When financial refunds result from product return, these can be used as one variable, product return. Similarly, other studies include product returns, claims, and maintenance or repair services as post-sale activity (Frasquet et al., 2016). Recent studies such as Darghout et al. (2017) suggest that customers’ purchase decisions are influenced not only by pre-purchase elements (i.e., product’s useful life, performance, and price), but also by post-purchase support service (i.e., repair). Thus, the dimensions of post-sale services included in this study are returns, product exchanges, and maintenance or repair.

**FIGURE 1:**
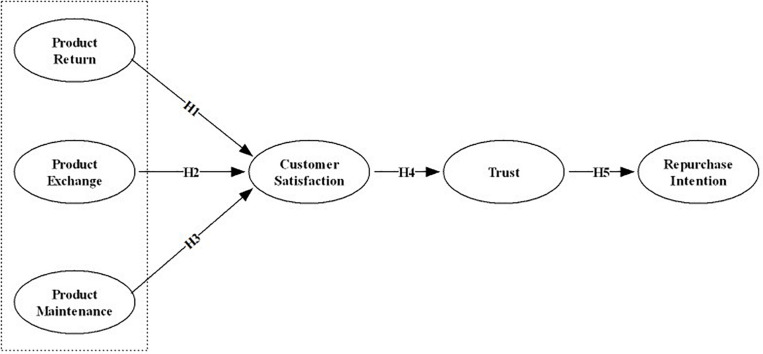
Hypothesized model.

Because the online shopping environment is considered competitive and complex, researchers such as Azadeh et al. (2017) have identified some factors that play a key role, and Giovanis and Athanasopoulou (2014) notes these to include a retailer’s ability to provide excellent services, quality customer-retailer relationships, and success in gaining repurchase or loyalty. In the view of Morgan and Hunt (1994), the act of maintaining and establishing relational exchanges represents a key change in marketing practice and theory, further stating that their theory applies to all relational exchanges connecting consumers and suppliers. Prior studies (Crosby et al., 1990; Morgan and Hunt, 1994; Wang and Kim, 2019) have conceptualized the effect of relationship marketing on consequences as completely mediated by one or both of the interpersonal constructs of satisfaction and trust. Research also points to satisfaction and trust as key determinants of long-term relationships in an online environment and customer repurchase intention (Agag and El-Masry, 2016; Al-Adwan and Al-Horani, 2019; Rita et al., 2019). Researchers further note that very few studies have focused on the customers’ attitudinal and behavioral aspects (Jain et al., 2017) in the context of online retailer’s post-sale services. Thus, we finalize our research or proposed model (Figure 1).

In addition, services provided in online shopping environments are related to a set of psychological dispositions (Lai et al., 2014), with motivational differences existing between females and males (Sangwan et al., 2009; Shi et al., 2018). For example, research suggested that compared to males, female customers consider a higher probability of negative consequences in the online shopping context (Garbarino and Strahilevitz, 2004). Prior research further suggests that female customers are more sensitive than males and more care about details related to the service provided by internet retailer (Shi et al., 2018). Such gender differences lead to different influences of e-commerce constructs on their online shopping decisions.

With respect to on-line clothing purchases, past studies reveal gender to be a more relevant variable than income or age level (Goldsmith and Flynn, 2004). Individuals’ personal characteristics thus have a key role in shaping their perceptions about an on-line company and in developing satisfaction and trust (Martín and Jimenez, 2011). Accordingly, researchers agree that in terms of swaying consumers’ online shopping behavior, gender should be considered as an important demographic variable (Goldsmith and Flynn, 2004; Kim et al., 2007; Wang and Kim, 2019). Consequently, a comparison of perceived post-sale services among female and male customers has been suggested, considering that it would enhance marketers’ and researchers’ understanding the post-sale services of an online retailer. Based on these findings, we will address the issue of online retailers’ post-sale services and develop managerial recommendations on how online retailers can improve their post-sale service management strategies, focusing on gender differences.

This study procedure has two steps. We first investigate the direct impact of product return, exchange and maintenance services on customer satisfaction and satisfaction’s influence on customer trust. We then examine the mediating effect of relationship quality (such as satisfaction and trust) regarding perceived product return, exchange, maintenance services, and customer repurchase intention. To achieve research objectives, hypotheses were developed and tested on the basis of extant literature. To conclude, the current study’s implications from both academic and managerial perspectives will be discussed as well as the limitations and future research directions.

### Returning of Purchased Products

Product return has been a prolonged challenge for online retailers (Kaushik et al., 2020). Product return results in a retailer returning or crediting consumer payment. Earlier, product return was primarily introduced to reduce the uncertainty of product quality as a competitive strategy, but now it has become an essential part of the transaction procedure (Han et al., 2017). Product return is more important in online shopping than offline because consumers have no opportunity to inspect the product physically (Dholakia et al., 2005) and must rely on photos and textual imageries that may not be sufficiently accurate (Fu et al., 2016). Consequently, making accurate purchase decisions is very difficult for online customers (Maity and Dass, 2014) and they may choose to return unsatisfactory products to the retailer as per the return policy (Fu et al., 2016). Thus, online retailers’ return policies can motivate or demotivate the customers to repurchase from the same retailer. Offering a return policy, by which consumers can return the purchased products for refund, has been used as a significant marketing tool and a competitive strategy to considerably increase consumer satisfaction and improve product sales (Mukhopadhyay and Setoputro, 2005). Similarly, a recent study suggested that as online shopping does not offer the ability to handle the product before purchase, a retailer’s return policy is a tool to enhance sales and boost loyalty (Oghazi et al., 2018).

Regardless of the return policy, customer support’s response during a call regarding returns may affect customers’ future purchasing based upon its role during an order return, simple procedures for refunds on an order, and the expressed time necessary for a refund after return. Customers are likely to experience difficulty in online purchasing (Lai et al., 2014) when they face insufficient customer service responsiveness and bad experiences with product return procedures (Cowles et al., 2002). However, satisfactory arrangements for product return are a significant element in the competitive market, and companies’ performance in managing product returns plays a significant role in swaying consumer loyalty or repurchases (Ramanathan, 2011).

### Product Exchanges

Exchange service provides online retailers one more chance to interact with customers (Han et al., 2017). Customer exchange decisions may stem from an issue with the product itself, the degree of brand matching, or the level of compatibility among the products to be replaced (Rahinel and Redden, 2013). Therefore, the consumer could opt to buy a different product/or one with the same, lower, or higher value after interacting with the retailer (Han et al., 2017).

Online retailers’ service failures at the product exchange stage may disappoint customers and affect their future purchases. Conversely, adequately dealing with product exchange through simple procedures or adherence to the expressed time taken for an exchange may positively affect future purchasing. Because the internet is an influential tool in reinforcing consumer bonds, the online sellers’ satisfactory responses in their dealings are helpful in increasing customer loyalty (Reichheld and Schefter, 2000). By the same token, the positive evaluation of an internet retailer’s services for exchanging a product may positively influence customer-retailer relationship quality, because the pleasure or convenience obtained from high efficiency service helps to foster a long-term customer-service provider relationship (Khan et al., 2014).

### Maintenance or Repair

Maintenance or repair of products purchased online is very important to customers especially for electronic products. For example, when purchasing an item such as a DSLR camera that is very complex with costly parts and components that may be easily broken after sale, service matters enormously (Chen et al., 2017). Similarly, home appliances and electronics items purchased through an online store may require repairs during the warranty period. Accordingly, after-sale service (such as maintenance or repair) can be provided by either the retailer or manufacturer based on a contract (i.e., consignment or/and wholesale contract) between the retailer and manufacturer (Chen et al., 2017).

Furthermore, base warranty, which is an after-sales service, is not only an obligatory condition by law but also a means to enhance competitiveness for businesses (Li et al., 2014). Such warranties provide opportunities to maintain and build a longer relationship with customers (Rezapour et al., 2016). For products based on increasingly sophisticated technologies, after-sale service (e.g., repairing) is becoming increasingly important (Owen and Mobin, 2015). Buyers often take it for granted and consider it as part of product offerings and it is commonly part of the retail price (Kranenburg and van Houtum, 2008). Thus, consumers’ valuation of a product can be enhanced using such offerings in order to build the customer-retailer relationship and the product’s perceived value (Falk and Hagsten, 2015).

Online retailers’ service level commitment must be guaranteed in order to stimulate demand (Li et al., 2014). However, retailers’ commitment to such services would be believable for customers when they can make their own evaluations or when they are informed of the actual service level (Allon and Federgruen, 2007). Therefore, customers’ experiences regarding actual service level should never be lower than the retailers’ commitment, but may sometimes be higher (Li et al., 2014). In this regard, online retailer policies to deal with repair matters, the time required to deal with the repair issues, and accordingly their satisfactory responses regarding the repair of products may matter for customers. The actual service level experienced by customers will influence their level of satisfaction in a retailer.

### Relationship Quality as Mediators

Relationship quality is commonly known as both an important predictor of customer post-purchase behavior (Morgan and Hunt, 1994) and a key to cultivating consumers’ loyalty (Walsh et al., 2010). Trust and satisfaction are considered the basic core dimensions of consumer market relationship quality (Shan et al., 2013). However, anything that might affect those relationships deserves research attention (Walsh and Möhring, 2017).

#### Customer Satisfaction

Customer satisfaction is essential to business longevity (Al-Adwan and Al-Horani, 2019). Customer satisfaction topics are therefore heavily emphasized by scholars, and accordingly studies have been conducted on post-consumption behavior such as loyalty or repurchase intention (Cooil et al., 2007). Satisfaction refers to consumers’ overall evaluation of the total buying and consumption experience with services or products over a time period (Anderson et al., 2004). E-satisfaction refers to the customers’ gratification with their previous buying experience on an e-shopping website (Moriuchi and Takahashi, 2016).

Researchers suggest that customers not only assess the products or services offered online but also evaluate their need for service support (i.e., post-sale services) (Wolfinbarger and Gilly, 2003). The customers’ service quality experience through the online service process leads them to form a judgment about online service satisfaction (Zhou et al., 2019). In view of that, Al-Adwan and Al-Horani (2019) commented that customers are satisfied if the perceived performance matches their expectations. Moreover, several studies revealed that e-service quality has a significant positive effect on customer satisfaction (Martín and Jimenez, 2011; Cao et al., 2018; Rita et al., 2019; Wang and Kim, 2019). Therefore, we hypothesized accordingly:

H1: Product returns have a positive impact on customer satisfaction.

H2: Product exchanges have a positive impact on customer satisfaction.

H3: Maintenance services have a positive impact on customer satisfaction.

### Customer Trust

Trust refers to a key component of retaining buyer-seller relationship (Chiu et al., 2012). An online shopping environment involves various risks (Moriuchi and Takahashi, 2016). To mitigate the effects of these risks facing consumers’ relationships (as shoppers and retailer) in the e-commerce environment, consumers therefore rely on their trust in the retailer (Ha and Stoel, 2008; Al-Adwan and Al-Horani, 2019). However, building consumer trust is a difficult procedure, and their involvement in online settings ranges from transactional to relational (Oghazi et al., 2018). Hence, for the success of e-commerce, trust is considered as one of the most crucial prerequisites (Hsu et al., 2016).

Several studies have suggested that trust is a key facet in online commercial transactions, particularly because of the complex social surroundings (McKnight et al., 2002; Gefen et al., 2003). In other words, individuals are likely to use trust as a key social complication deduction approach when the social environment is away from the control of red tape (Luhmann, 1979). This seems a valid argument in the context of online environments, as online consumers tend to trust a retailer who is credible and upholds socially acceptable behavior. Gefen (2000) further explained that if online retailers do not demonstrate socially responsible attitudes, customers facing the problematic social complexity will finally avoid engaging with them regarding online buying. Therefore, satisfactory online experiences are expected to lead to customer e-trust (Helgesen and Nesset, 2010; Al-Adwan and Al-Horani, 2019). In the same vein, Martín and Jimenez (2011) suggest that satisfaction with prior experience is a key aspect in generating trust. In such circumstances, studies further confirm that e-satisfaction significantly influences e-trust (Martín and Jimenez, 2011; Shin et al., 2013; Hung et al., 2019). Therefore, we hypothesized accordingly:

H4: Customer satisfaction has a positive direct effect on customer trust.

### Repurchase Intention

Repurchase intention in online shopping is defined as customer willingness to buy products or services again in the future from an online retailer’s website (Chauke and Dhurup, 2017). Intent to repurchase is considered a reflection of customer loyalty according to marketing literature (Gruen et al., 2006). Compared to other dimensions of loyalty, intent to repurchase is a dependable psychological forecaster of repeat purchase behavior, and such conduct will have a direct effect on a retailer’s profit (Crosby et al., 1990). Repurchase intention is very important for corporate success because the cost of retaining an existing customer is very low compared to finding and serving a new one (Spreng et al., 1995; Javed et al., 2019). Retailers therefore have to offer high-quality services to their service recipients if they desire to motivate them into loyalty (Keng et al., 2007). Studies indicate that customers who are satisfied will not only build trust but will also cultivate more positive intentions and thus purchase more from the firm (Anderson and Mittal, 2000). Thus, trust is positively associated with intent to repurchase (Lee et al., 2011; Shin et al., 2013; Rita et al., 2019; Al-Adwan and Al-Horani, 2019). Therefore, we hypothesized accordingly:

H5: Customers’ trust has a positive direct effect on their intent to repurchase.

## Methodology

### Measurement Development

Previous research-based measurement items were modified and translated to Chinese to better fit this study’s context. A five point Likert scale ranging from 1-strongly disagree to 5-strongly agree was used to measure the construct items.

The dimensions of post-sale services—product return, exchange, and maintenance/repair—were measured using three modified items from Jeng (2017) and Lai et al. (2014). Customer satisfaction was measured using three items modified to serve this study’s purpose, and these were adopted from Wu (2013) and Deng et al. (2010). Moreover, the items for measuring trust were adopted from Gefen et al. (2003), whereas three items used to measure repeat purchase intention were based on Parasuraman et al. (2005).

### Survey Administration

Using jd.com (a leading online retail store in China particularly famous for the sale of electronic products) as a data source, data were collected for four digital product categories (i.e., mobile phones, computers, cameras, and digital watches – see Table 1). Digital goods are different from traditional goods in their intangibility and are distributed directly through website (Laroche et al., 2001). Simultaneously, intangibility has a higher influence on perceived risks associated with a product particularly in the case of services (Laroche et al., 2005), functionality, and durability. In such circumstances, online retailers’ post-sale services are not only considered helpful to mitigate perceived product risks and encourage customers to revisit the store. Moreover, only those respondents purchasing products from jd.com in last 1 year and who have used the post-sale services for their purchased goods will be included in the final sample.

**TABLE 1:** Respondents’ demographics and product categories.

Age	Frequency	Education	Frequency	Gender	Frequency	Products categories			
						Mobile phones	Computers	Cameras	Digital watches
<18	4	High school	33	Female	221	3			1
18–25	182	Bachelor	249	Male	188	92	50	10	30
26–33	93	Master degree	112			38	26	11	18
34–41	79	Ph.D. or above	15			35	10	22	12
42–49	35					25	1	9	
≥50	16					10		6	

Before sending out the questionnaires, a pretest was conducted (*n* = 20) to ensure the correctness of the questionnaire’s wording, constructs assessment, and statistical standards. An online questionnaire was used to collect data for jd.com post-sale services throughout China. Online surveys can be more effective for reaching and identifying online shoppers (Elbeltagi and Agag, 2016), and an online approach thus offered a more convenient and efficient data collection form (Best and Krueger, 2002). Accordingly, a total of 442 participants responded to an online survey questionnaire.^1^ A final sample of 409 respondents who used the post-sale services of jd.com was included for further processing after eliminating the incomplete questionnaires (*n* = 18) and those who chose the options of never purchased from jd.com (*n* = 9) or never used post-sale services (*n* = 6) in the last 1 year.

## Results

### Demographic Profile

About 46% of the respondents were male, a majority of the participants were between the ages of 18 and 25, and 61% of the participants had a bachelor’s degree, all of which improved the sample pool’s level of heterogeneity. A detailed overview of the demographic sample is presented in Table 1.

### Measurement Results

Six factors and 18 items of the measurement model were estimated with Amos software. We analyzed the overall goodness of fit [χ^2^ (120) = 316.533, *P* < .000; normed Chi-square χ^2^/*df* = 2.637; and the alternative fit indices, i.e., GFI = 0.924; AGFI = 0.896; CFI = 0.977, NFI = 0.959, RMSEA = 0.067, and PCFI = 0.813] and accepted the model (Table 2). Further, composite reliability (CR) and Cronbach’s α were used to assess internal consistency. The values of Cronbach’s α and CR of coefficients were found all above 0.70 standards (Fornell and Larcker, 1981). Thus, the items in the questionnaire were reliable (See Table 3).

**TABLE 2:** Model fit indicators.

Model fit indicators	Benchmark	Model values
Normed Chi-square χ^2^/*df*	≦3.00	2.637
Goodness-of-fit index (GFI)	≧0.90	0.924
Adjusted goodness-of-fit index (AGFI)	≧0.80	0.896
Comparative fit index (CFI)	≧0.90	0.977
Normed fit index (NFI)	≧0.95	0.959
Root mean square error of approximation (RMSEA)	<0.08	0.067
Parsimony comparative fit index (PCFI)	≧0.80	0.813

**TABLE 3:** Reliability indices for constructs.

Variable	CR	Cronbach’s α
Product return	0.8577	0.856
Product exchange	0.8804	0.879
Product maintenance	0.8796	0.878
Customer satisfaction	0.8696	0.869
Customer trust	0.8968	0.893
Repurchase intention	0.8755	0.872

To test construct validity, the confirmatory factor analysis (CFA) is widely uses as an effective tool. Usually, the degree of data availability (i.e., convergent validity and discriminant validity) is tested using construct validity (Campbell and Fiske, 1959). Table 4 indicates a strong convergent validity and statistical significance as all loading factors and the average variance (extracted from items) are over the benchmark of 0.5 (He and Li, 2011).

**TABLE 4:** Convergent validity.

Construct	Measures	Loading	AVE
Product return			0.6682
Return1	As per my perception, an adequately fair time was taken for product returns by this website	0.814	
Return2	As per my perception, conditions stated by this website for product returns were flexible	0.870	
Return3	As per my perception, the overall procedure of product returns is convenient	0.765	
Product exchange			0.7107
Exch1	As per my perception, an adequately fair time was taken for product exchanges by this website	0.840	
Exch2	As per my perception, conditions stated by this website for product exchange were flexible	0.873	
Exch3	As per my perception, the overall procedure of exchanges is convenient	0.815	
Product maintenance			0.7092
Maint1	As per my perception, an adequately fair time was taken for product repair by this website	0.839	
Maint2	I observed that the time taken by this website for product maintenance/repair was adequately fair	0.878	
Maint3	As per my perception, the overall procedure of product repair is convenient	0.808	
Customer satisfaction			0.6898
Satisf1	By buying from this website, I think I did the right things	0.818	
Satisf2	On this website, I have really enjoyed myself	0.856	
Satisf3	As per my overall experience with this website, I am very satisfied	0.817	
Customer trust			0.7435
Trust1	As per my past experience, I know that this website cares about its customers	0.855	
Trust2	As per my past experience with this website, I know that it is not opportunistic	0.841	
Trust3	As per my past experience, I know that this website keeps its promises to its customers	0.890	
Repurchase intention			0.7017
Repur1	If I could, I would like to continue using this website to purchase products	0.868	
Repur2	There is a strong likelihood that I will continue purchasing products from this website in the future	0.872	
Repur3	If current services are continued, I am tending to buy the products I need continuously at this store	0.769	

As Table 5 shows, the discriminant validity was checked to assess the correlation matrix. We found that the square root of AVE as diagonal elements is more than the off-diagonal elements (inter-construct correlations coefficient). It means that each construct shared more variance with its items than it did with other constructs. In conclusion, these results prove the validity and reliability of our current model. In order to address the issues of common method bias (CMB), a CFA approach to the Harman method was applied as it is considered more classy than exploratory factor analysis (Podsakoff et al., 2003). Accordingly, a CFA model was computed that constrained the factors and items used in the current study to load on a single factor. A very poor model fit such as χ^2^ (119) = 987.63, *P* = 0.000; normed Chi-square χ^2^/*df* = 8.299; and the alternative fit indices, i.e., CFI = 0.73; RMSEA = 0.13; GFI = 0.67; AGFI = 0.61; and PCFI = 0.47; revealed that CMB was not an issue.

**TABLE 5:** Discriminant validity of the constructs, mean, and standard deviation.

	Mean	SD	Repurchase	Trust	Maintenance	Exchange	Satisfaction	Return
Repurchase	3.893	0.756	**0.837**					
Trust	3.843	0.717	0.805	**0.862**				
Maintenance	3.594	0.820	0.690	0.767	**0.842**			
Exchange	3.741	0.739	0.620	0.728	0.819	**0.843**		
Satisfaction	3.768	0.758	0.751	0.845	0.812	0.765	**0.830**	
Return	3.524	0.811	0.558	0.640	0.749	0.750	0.708	**0.817**

*SD is Standard deviation, while bold items are the square root of the average variance extracted.*

### Structural Relationship or SR Results

For path analysis (how constructs actually relate to each other) and to test the hypotheses relationship, we use the SR model. The SR model results exhibited acceptable fit as the χ^2^ = 345.583(130), *P* < 0.000, normed Chi-square χ^2^/*df* = 2.658, and the alternative fit indices, that is CFI (0.977), NFI = (0.961), GFI (0.925), AGFI (0.896), PCFI (0.814), and RMSEA (0.067), were found statistically significant.

The post-sale services dimensions such as product returns (β = 0.197, *t* = 3.743), exchanges (β = 0.333, *t* = 5.716), and maintenance (β = 0.476, *t* = 8.253) were revealed as important antecedents in determining the effects of post-sale services on customer satisfaction in the online shopping environment. Consequently, H1, H2, and H3 are supported.

In favor of H4, the relationship between satisfaction and customer trust is also supported (β = 0.848, *t* = 19.184). This relationship shows that customers’ satisfaction has a direct influence on their trust level. For H5, the analysis also provides support (β = 0.878, *t* = 17.474). This indicates that customer trust has a direct significant effect on repurchase intentions. Table 6 presents the detailed results of hypotheses relationships.

**TABLE 6:** Hypotheses results.

Hypothesis	Estimate	*t*-Value	Support
Product return → satisfaction	0.197	3.743***	H1: Yes
Product exchange → satisfaction	0.333	5.716***	H2: Yes
Maintenance → satisfaction	0.476	8.253***	H3: Yes
Satisfaction → customer trust	0.848	19.184***	H4: Yes
Customer trust → repurchase	0.878	17.474***	H5: Yes

***P < 0.001.

**FIGURE 2:**
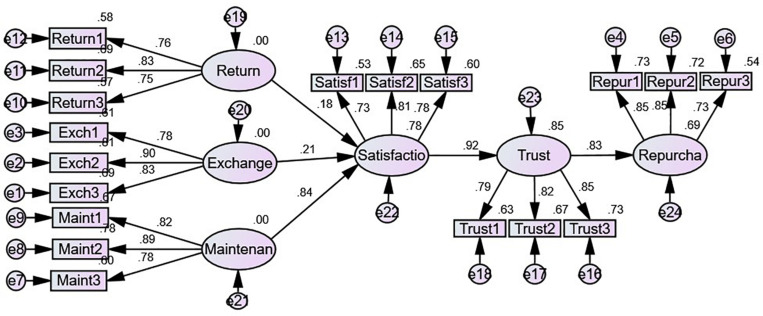
Results for male (gender code = 1) sample.

**FIGURE 3:**
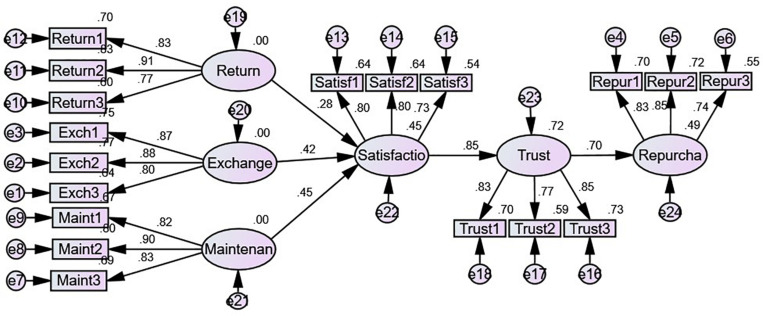
Results for female (gender code = 2) sample.

In order to further analyze whether there are differences between males and females in the above SR model, we make a supplementary analysis. We first split the sample according to gender, then conduct analyses using Amos. The results are shown in Table 7 and Figures 2, 3.

**TABLE 7:** Results for male and female sample.

	Male			Female		
	Estimate	*t*-Value	Support	Estimate	*t*-Value	Support
Return → satisfaction	0.166	2.257*	H1: Yes	0.232	3.115**	H1: Yes
Exchange → satisfaction	0.179	2.315*	H2: Yes	0.383	4.441***	H2: Yes
Maintenance → satisfaction	0.702	7.226***	H3: Yes	0.375	4.830***	H3: Yes
Satisfaction → trust	0.916	12.942***	H4: Yes	0.767	14.124***	H4: Yes
Trust → repurchase	0.896	13.441***	H5: Yes	0.840	11.144***	H5: Yes

***P < 0.001; **P < 0.01; *P < 0.05.

In general, for men and women, the mechanism of post-sale services affecting repurchase intention is the same. That is, post-sale services affect repurchase intention through customer satisfaction and trust. In terms of specific differences, for male samples, as shown in Table 7 and Figure 2, it seems they are more concerned with maintenance/repair (β = 0.702, *P* < 0.001) than return (β = 0.166, *P* < 0.05) or exchange (β = 0.179, *P* < 0.05). At the same time, for the male sample, the direct effect between customer satisfaction and trust (β = 0.916, *P* < 0.001) and trust on repurchase is also significant (β = 0.896, *P* < 0.001). For the female sample, as shown in Table 7 and Figure 3, it seems that they are more concerned about exchange (β = 0.383, *P* < 0.001) and maintenance (β = 0.375, *P* < 0.001) in post-sale services than return (β = 0.232, *P* < 0.01). The direct effect between customer satisfaction and trust (β = 0.767, *P* < 0.001) and trust on repurchase intention is also significant (β = 0.840, *P* < 0.001) for females. In conclusion, for the post-sale service issue, men pay more attention to maintenance, whereas women pay more attention to both exchange and maintenance/repair.

## Discussion

Online retailers’ ability to attract and retain customers is vital for the success of every business. Repurchase or customer loyalty requires the buyer’s strong need for a product and the buyer’s choice of a product based on his/her preferences among several product vendors (Dick and Basu, 1994; Otim and Grover, 2006). In this context, the quality of post-sale services may be important to distinguish an online retailer from others and to attract returning shoppers (Pee et al., 2018). Because these services are not only helpful to reduce customers’ uncertainty regarding a retailer (Ramanathan, 2011), they may also be considered as security for the customer against their online shopping. However, prior research into online retailers’ post-sale services was limited to addressing customer perceptions and motivations regarding online retailers’ product return (Javed and Wu, 2020). Therefore, the current study focuses on shedding light on the role of all post-sale service dimensions (i.e., product exchange, return, and maintenance/repair services) in developing customer repurchase intention.

Research has also suggested that gender may be one of the most distinctive differences in consumer innovativeness studies due to their different roles in society (Kim et al., 2011). Accordingly, the purchase patterns and behaviors of female and male customers show noteworthy variances (Chang and Yeh, 2016). However, there is no information in the online retailer’s post-sale service literature indicating whether male and female have different perceptions about product return, exchange, and maintenance when they shop online. Accordingly, a comparison between male and female online customers was suggested.

The proposed model in this study thus examines the effect of product return, exchange and maintenance/repair services on customer repurchase intention through the mediating effect of relationship quality between perceived post-sale services from online retailers and their subsequent effect on customer repurchase intention in an online shopping environment. The outcomes of this research indicate that product return, product exchange, and product maintenance all have positive, direct effects on customer satisfaction. Positive perceptions of an online retailer’s post-sale services may enhance consumers’ online shopping experiences, which in turn shapes their satisfaction with a retailer. This is consistent with prior research by Martín and Jimenez (2011); Rita et al. (2019), and Wang and Kim (2019), who revealed that service quality has a direct influence on customer satisfaction.

Notably, exchange (β = 0.333) and maintenance (β = 0.476) services exerted the strongest effect on customers’ satisfaction of the three dimensional structure of post-sale services. This was followed by product return (β = 0.197). The possible reason may be that product exchange and maintenance are not as simple as product return, and customers are more concerned about product exchange and maintenance. This notable finding reveals the important role of product maintenance and exchange in the context of online retailers’ post-sale services in developing customer satisfaction.

Customer satisfaction further mediates the relationship between the dimensions of post-sale services and customer trust. Results indicate that satisfaction with prior experiences is an important aspect in generating trust. This outcome is consistent with prior research (e.g., Martín and Jimenez, 2011; Hung et al., 2019; Wang and Kim, 2019) that confirmed satisfaction’s mediating role between e-service quality and customer trust in an online shopping context.

Both customer satisfaction and trust mediate the relationship between product return, exchange, maintenance, and repurchase intention. This demonstrates that post-sale services are not only a significant determinant of customer-retailer relationship, but also lead to an increase in sales. Thus, online retailers’ post-sale services are proven as one of the notable factors in sustaining consumers in online shopping platform and directly influencing their value perceptions. This also reveals that customers’ concrete usage practices change perceptions of convenience and shape the validation of their initial requirements (Lãzãroiu et al., 2020).

In terms of specific differences, for the male sample, product maintenance exerted a higher direct effect on customer satisfaction (β = 0.702, *P* < 0.001), whereas product exchange (β = 0.179, *P* < 0.05) and product return (β = 0.166, *P* < 0.05) had relatively lower direct influence. This is likely due to male customers being more goal oriented and caring more about the efficiency of online purchase (Shi et al., 2018). Accordingly, product maintenance or repair features are more important among male online shoppers.

In contrast to the male sample, both product exchange (β = 0.383, *P* < 0.001) and maintenance (β = 0.375, *P* < 0.001) had a positive higher effect on customer satisfaction among online female customers, followed by product return (β = 0.232, *P* < 0.01). One possible explanation for this is that female customers weigh product exchange and maintenance more heavily in the context of online retailers’ post-sale services.

While analyzing both male and female samples, it was found that product repair and maintenance service is the only dimension of online retailers’ post-sale services having a strong direct effect on customer satisfaction. One possible reason for this is that maintenance is becoming increasingly important for products due to their sophisticated technologies. This reflects another important contribution by the current research to the literature on online retail overall and particularly on online retailers’ post-sale services.

## Managerial Implications

Competition, needs, and customer behaviors have all become very significant to service providers (Azadeh et al., 2017). Research has suggested that post-sale activities play an important role for online retail businesses because they are an opportunity to attain customer satisfaction and retention (Frasquet et al., 2016). Therefore, in order to improve the efficiency of online services, this research recommends several influencing factors. Firstly, the results revealed positive direct effects of product return, product exchange, and maintenance or repair services on customers’ satisfaction. These results indicate that post-sale services from online retailers are important determinants in cumulative satisfaction among online shoppers. Zhou et al. (2019) highlighted how customers’ experiences of service quality through the online service process leads them to form judgments about online service, and customers are satisfied if the perceived performance matches their expectations (Wilson et al., 2012). Giving importance to online retailers’ post-sale services, Rajendran et al. (2018) said that online retailers should focus on the post-purchase consumer experience at a maximum level to convert them into satisfied customers. In contrast, inefficient post-sale services may lead to online customers’ retaliation (Arruda Filho and Barcelos, 2020). Therefore the success of online business will greatly depend on improving procedures related to exchanging, returning (Hong and Cha, 2013), and repairing purchased products.

The results of the main model confirmed the stronger influence of product exchange and maintenance on customer satisfaction. Product exchange and maintenance are not as simple as product return, yet customers assign this a significantly higher value. For example, with product exchanges, the customer has to wait twice when ordering a new product compared to the time if the original purchase had met their expectations. In order to manage product exchange efficiently, it will be beneficial to inform customers about the expected date for receiving an exchange. For maintenance/repairs, the customer may have to send the product back to the retailer or take it to the assigned repair dealer and must understand what is covered or not covered by warranty. The maintenance/repair-level commitment must therefore be guaranteed to stimulate demand. Higher service levels provided to customers by a retailer correspond to greater sales (Li et al., 2014). Firms must thus exercise continuous control to avoid discrepancies between what was promised in the pre-sale phase and what was finally accomplished and resolved in the post-sale phase (Alzola and Robaina, 2010).

The connections between customer satisfaction and trust, online retailers’ post-sale services, and customers’ repurchase intention indicates that better management of product return, exchange and maintenance/repair services improves feelings of association and cultivates a longer associative relationship. It suggests that the pleasure obtained from high-efficiency product returns, exchanges, and maintenance helps foster a long-term customer-retailer relationship. In this regard, research has noted that poor customer relationship management does not kill, whereas good customer relationship management adds profit (Stone, 2011). Thus, like product returns, product exchanges and maintenance services not only have an important role in building the customer-retailer relationship but also affect customer repurchase behavior. Prior studies indicate that when customers feel satisfied with and have trust in an online store, it may reinforce their intent to repurchase (Chou et al., 2015). In the same vein, Rajendran et al. (2018) empathize that a customer’s positive post-sale experience will result in customer repurchase intention. Therefore, online retailers should also give importance to product exchanges and maintenance together with product return when making decisions about post-sale service policies and the according allocation of resources.

All three dimensions of online retailers’ post-sale services have a direct effect on customer satisfaction, whereas trust further mediates the relationship between customer satisfactions and repurchase intention. Studies suggest that satisfaction is defined as customers’ gratification with their prior purchasing experience (Moriuchi and Takahashi, 2016), whereas trust reflects the consumers’ confidence in an online retailer’s upcoming performance (Zhang et al., 2011). In the context of these explanations, the quality of post-sale services is not only a remarkable factor in reducing online shopping uncertainty (Hong and Cha, 2013), but also as a way to sustain customers by offering a frictionless and pleasurable shopping experience (Chen et al., 2017; Singh, 2019). Moreover, to boost an online retailer’s credibility and to reduce the online shopping environment’s risks, posting more information about the online retailer’s business history can help customers gain confidence in the retailer.

In order to plan more effective marketing strategies, marketers therefore should seek to identify the different ways in which males and females think with regard to their purchases (Tifferet and Herstein, 2012; Shi et al., 2018; Teeroovengadum, 2020). Accordingly, the outcomes of this research show that male customers give more weight to maintenance services in the context of online retailers’ post-sale services. Hence, among other services, male customers not only pay close attention to an online retailer’s product maintenance or repair policy but also want easy access to a repair outlet. Prior research also suggests that male customers may show greater satisfaction and trust in those retailers who offer a warranties or repair services (Martín and Jimenez, 2011). To secure future loyalty, online retailers must not only make repairs easily accessible, but also guarantee their repair-level commitment.

In evaluating an online retailer’s post-sale service, female consumers indicate that product exchange and maintenance services are most important. Product exchange normally takes more time, and customers have to wait for at least a week before receiving an exchanged item. Prior researchers found that the waiting time is one of the main concerns for female online customers (Chou et al., 2015). Therefore online retail managers should use tracking systems for exchanged products to increase convenience and avoid a stressful wait (Lai et al., 2014). With regard to maintenance services, Owen and Mobin (2015) commented that after-sale repair services have risen in importance due to increasingly sophisticated technologies. Consequently, the repair-level commitment must be guaranteed in order to build customer-retailer relationship and to encourage customer repurchases.

Online retailers provide order fulfillment or product reviews on their website, but there is no column for customer post-sale experiences such as return, exchange, or maintenance. Our study has revealed that product return, exchange, and maintenance have the potential to generate customer intent to repurchase, and thus reviews of customers’ post-sale experiences could be a valuable addition to online retailers.

Post-sale services such as product return, exchange and maintenance are also an ethical matter because these activities do not add profit for the online retailer at this stage. However, current research has proven that these have great significance not only in building customer-retailer relationships but also in influencing future purchases. Prior research also noted that customers also consider the seller’s honesty and responsible attitude when they shop (Bussey, 2006). Thus, an online retailer’s history of responsibly post-sale services can generate new future business.

In the online shopping context, factors from the employees’ behavioral perspective, such as processing speed, execution quality, confusion from analogous color, and disturbance from task-switching, deserve more attention. Therefore, front line staff often play an important role in delivering customer satisfaction (Homburg et al., 2009). Front line staff quality is generally assessed according to customer post-purchase valuation views (Jones, 2014). Moreover, a service failure can be turned into service delight by a skilled team (Ramanathan, 2011). For continuously high quality performance by employees, retail companies need to focus on their employees’ regular training sessions and provide bonuses based on performance.

## Limitations and Future Research Directions

Prior research has paid little attention on online retailers’ post-sale services as a remedy to online shopping uncertainty. Taking an inclusive approach to tackle the uncertainty of online buying, this study integrates three dimensions of online retailers post-sale services (return, exchange, and maintenance) linked with relationship quality and revisit intention. Our study sheds further light on the literature through the lens of male and female online customer preference, and its research approach can serve as a basis for future studies examining online business success. However, the outcomes of the current research should be interpreted within context and with caution. Because our sample consisted mainly of Chinese online customers, the gathered responses may not represent attitudes in other countries. However, this study’s model can examined with different data collected from other countries. Secondly, collecting online data is effective for reaching customers all around a country, but it might introduce some bias into the outcomes due to this population’s differing motivations. Additionally, data was collected only for one store,^2^ which has warehouses in all of China’s big cities. They are very quick at initial delivery; however, their post-sale services might not be so quick. Thus, customers’ opinions might be based on this difference. Future studies therefore need to consider more than one online store in order to obtain customers’ responses regarding post-sale services. Moreover, a comparison among different online websites or stores would also have interesting results to better manage an online retailer’s post-sale service. Product category might also be an important factor in measuring customers’ beliefs about a retailer’s post-sale service, but this research lacks the capacity to explore this factor. Further research may incorporate product category to have a closer look at post-sale services.

## Data Availability Statement

The raw data supporting the conclusions of this article will be made available by the authors, without undue reservation.

## Author Contributions

MJ developed the conceptual notions and drafted the manuscript. TQ and AM contributed in literature, methods, and analysis. MW and AN reviewed the manuscript critically, provided substantial contributions, and approved the final version to be submitted. RS edited the revised manuscript. All authors contributed to the article and approved the submitted version.

## Conflict of Interest

The authors declare that the research was conducted in the absence of any commercial or financial relationships that could be construed as a potential conflict of interest.

## Footnotes

1 www.wjx.cn

2 jd.com
